# Associations between polymorphisms of long non-coding RNA MEG3 and risk of colorectal cancer in Chinese

**DOI:** 10.18632/oncotarget.7764

**Published:** 2016-02-26

**Authors:** Xiangming Cao, Shulin Zhuang, Youfang Hu, Lei Xi, Lichun Deng, Huaming Sheng, Weisheng Shen

**Affiliations:** ^1^ Department of Oncology, The Affiliated Jiangyin Hospital of Southeast University Medical College, Wuxi, China; ^2^ Pukou District Center for Disease Control and Prevention, Nanjing, China; ^3^ Department of Children's Health, The First Affiliated Hospital of Nanjing Medical University, Nanjing, China

**Keywords:** lncRNA, MEG3, colorectal cancer

## Abstract

Maternally expressed gene 3 (*MEG3*), a long non-coding RNA (lncRNA), is involved in cancer development and metastasis. The objective of the present study was to evaluate whether common single nucleotide polymorphisms (SNPs) in *MEG3* could be related with colorectal cancer risk in Chinese. We genotyped six tagSNPs of *MEG3* in a colorectal cancer case-control study including 518 cases and 527 control subjects. Multivariate logistic regression analysis was applied to calculate adjusted odds ratios (ORs). We found that *MEG3* rs7158663 AA genotype, but not GA genotype, had significant increased colorectal cancer risk, compared with GG genotype (OR = 1.96 and *P* = 0.006 for AA versus GG, and OR = 1.20 and *P* = 0.171 for GA versus GG). Further stratified analysis indicated that the increased risk was significantly correlated with individuals with age ≤ 60 and family history of cancer. However, there was no significant association between rs7158663 and colorectal tumor site and stage (*P* = 0.842 for tumor site, and *P* = 0.601 for tumor stage). These results demonstrate that genetic variants in *MEG3* may contribute to the development and risk of colorectal cancer. Further studies are required to confirm these findings.

## INTRODUCTION

Colorectal cancer is the third most commonly diagnosed cancer in the world [1]. According to recent reports, the incidence and mortality of colorectal cancer in China have also been increasing in last two decades [2]. It is well known that colorectal cancer susceptibility is related to multiple environmental factors and genetic alterations, such as genetic mutations or polymorphisms [3, 4]. Nevertheless, the role of genetic polymorphism and colorectal cancer susceptibility still remains unknown.

Long non-coding RNAs (lncRNAs) are non-coding molecules larger than 200 nucleotides lacking significant protein-coding capacity [5]. It has been reported that lncRNAs are involved in diverse functions in carcinogenesis, including chromatin modification, transcription, and posttranscriptional processing [6]. In addition, previous studies have indicated that polymorphisms in lncRNAs may influence the risk of gastric cancer [7, 8]. Recent studies have demonstrated that lncRNA maternally expressed gene 3 (*MEG3*) is abnormal expressed in various human cancers, such as hepatocellular carcinoma [9, 10], bladder cancer [11], glioma [12], and gastric cancer [13]. Moreover, *MEG3* overexpression induced cell proliferation and was associated with the development and progression of colorectal cancer [14]. However, little is known about the single nucleotide polymorphisms (SNPs) in *MEG3* and colorectal cancer risk.

On the basis of previous findings mentioned above, as well as the influence of SNPs on *MEG3*, we hypothesis that genetic variants of *MEG3* may modify the development of colorectal cancer. The genetic variants of *MEG3* may be associated with the expression of *MEG3* and consequently influence susceptibility to colorectal cancer. To test the hypothesis, we carried out an association study between tagging SNPs (tagSNPs) in *MEG3* and colorectal cancer risk in a hospital-based colorectal cancer case-control study comprising 518 patients and 527 control subjects from China.

## RESULTS

The demographic characteristics of participants are described in Table 1. The average age of the patients was 60.0 years old compared with 59.2 years old in controls, which revealed no statistically difference (*P* = 0.284). Furthermore, there was no significant difference in sex distribution (*P* = 0.972) or smoking status (*P* = 0.292). However, the cases were asked to report a significant high rate of family history of cancer than the controls (*P* < 0.001). Among 518 patients, the number of cases with colon cancer and rectal cancer were 248 (47.9%) and 270 (52.1%), respectively. The tumor stage for I, II, III, and IV were 38 (7.3%), 214 (41.3%), 179 (34.6%), and 87 (16.8%), respectively.

**Table 1:** Distribution of characteristics among cases and controls

Variable	Cases (*n* = 518)	Controls (*n* = 527)	*P*
	*N* (%)	*N* (%)	
Age (mean ± SD)^a^	60.0 ± 12.4	59.2 ± 9.4	0.284
Gender			
Male	320 (61.8)	325 (61.7)	0.972
Female	198 (38.2)	202 (38.3)	
Smoking status			
Non-smokers	347 (67.0)	369 (70.0)	0.292
Smokers	171 (33.0)	158 (30.0)	
Family history of cancer			
No	405 (78.2)	488 (92.6)	< 0.001
Yes	113 (21.8)	39 (7.4)	
Site			
Colon	248 (47.9)		
Rectum	270 (52.1)		
Stage			
I	38 (7.3)		
II	214 (41.3)		
III	179 (34.6)		
IV	87 (16.8)		

a SD, standard deviation

The primary information of six SNPs in *MEG3* is summarized in Table 2. All SNPs in both cases and controls showed a call rate > 97.0%. The genotype frequencies in controls were in line with the Hardy-Weinberg equilibrium model (*P* = 0.712 for rs3087918, *P* = 0.930 for rs11160608, *P* = 0.812 for rs7158663, *P* = 0.521 for rs4081134, and *P* = 0.221 for rs10144253).

**Table 2:** Association analyses between SNPs in *MEG3* and colorectal cancer risk

SNP	Position	MAF in casea	MAF in control^a^	Call rate	HWE in case	HWE in control	OR (95%CI)^b^	*P*^b^
rs3087918	101297963	0.367	0.381	100.0%	0.344	0.712	0.94 (0.79-1.12)	0.493
rs11160608	101313093	0.433	0.436	100.0%	1.000	0.930	0.98 (0.82-1.16)	0.788
rs7158663	101319424	0.295	0.242	98.9%	0.138	0.812	1.31 (1.08-1.59)	0.007
rs4081134	101321788	0.227	0.219	97.8%	0.314	0.521	1.04 (0.84-1.29)	0.711
rs10144253	101325962	0.470	0.472	99.8%	0.659	0.221	0.98 (0.82-1.17)	0.843

a Minor allele frequency.

b Additive model adjusted for age, sex, and smoking status.

As shown in Table 2, the allele frequency of rs7158663 exhibited a significant difference between case and control groups (*P* = 0.007). The minor allele frequency (MAF) of rs7158663 in cases and controls were 0.295 and 0.242, respectively. However, no significant association with colorectal cancer was identified for other five SNPs (*P* = 0.493 for rs3087918, *P* = 0.788 for rs11160608, *P* = 0.711 for rs4081134, and *P* = 0.843 for rs10144253). In addition, after adjusting for multiple testing using Bonferroni correction, rs7158663 was still significant (*P* = 0.035).

We further performed a multivariate logistic regression analysis for rs7158663 by adjusting age, sex, and smoking. As listed in Table 3, rs7158663 AA genotype, but not GA genotype, had a significantly elevated risk of colorectal cancer, compared with GG genotype (*P* = 0.007) (OR = 1.96 and *P* = 0.006 for AA versus GG, and OR = 1.20 and *P* = 0.171 for GA versus GG). For genetic models, rs7158663 was significant between cases and controls in dominant, recessive, and additive models (*P* = 0.035, 0.012, and 0.007, respectively).

**Table 3:** Genotype frequencies of *MEG3* rs7158663 among cases and controls and their association with colorectal cancer risk

Genotype	Cases (*n* = 516)	Controls (*n* = 517)	OR (95%CI)a	*P*^a^
	*N* (%)	*N* (%)		
GG	264 (51.2)	298 (57.6)	1.00	
GA	200 (38.7)	188 (36.4)	1.20 (0.92-1.56)	0.171
AA	52 (10.1)	31 (6.0)	1.96 (1.22-3.17)	0.006
Dominant			1.31 (1.02-1.67)	0.035
Recessive			1.82 (1.14-2.91)	0.012
Additive			1.31 (1.08-1.59)	0.007

a Adjusted for age, sex, and smoking status.

We next evaluated the stratified association of rs7158663 with colorectal cancer risk by age, sex, family history of cancer, and smoking habits (Table 4). We observed that the GA/AA genotype hadan increased risk of colorectal cancer (OR = 1.31, *P* = 0.035). This increased effect was also more evident in subgroups of age ≤ 60 (OR = 1.71, *P* = 0.003) and patients with family history (OR = 1.25, *P* = 0.011). Nevertheless, no significant evidence was found for interaction between rs7158663 and these two factors.

**Table 4:** Stratified analyses for *MEG3* rs7158663 genotypes in cases and controls

Variable	GG	GA/AA	OR (95%CI)^a^	*P*^a^
	*N* (case/control)	*N* (case/control)		
Age (y), median				
≤60	104/158	124/111	1.71 (1.20-2.44)	0.003
>60	160/140	128/108	1.04 (0.74-1.46)	0.844
Gender				
Male	167/177	152/142	1.15 (0.84-1.57)	0.396
Female	97/121	100/77	1.62 (0.98-2.34)	0.076
Smoking status				
Non-smokers	168/207	178/155	1.42 (0.96-1.87)	0.062
Smokers	96/91	74/64	1.10 (0.70-1.71)	0.679
Family history of cancer				
No	204/268	200/210	1.25 (0.96-1.63)	0.099
Yes	60/30	52/9	2.98 (1.28-6.92)	0.011

a Adjusted for age, sex, and smoking status.

We evaluated the correlations between rs7158663 and clinical features of colorectal cancer, including tumor site and stage. As shown in Table 5, no significant relationship was observed (*P* = 0.842 for tumor site, and *P* = 0.601 for tumor stage).

**Figure 1:**
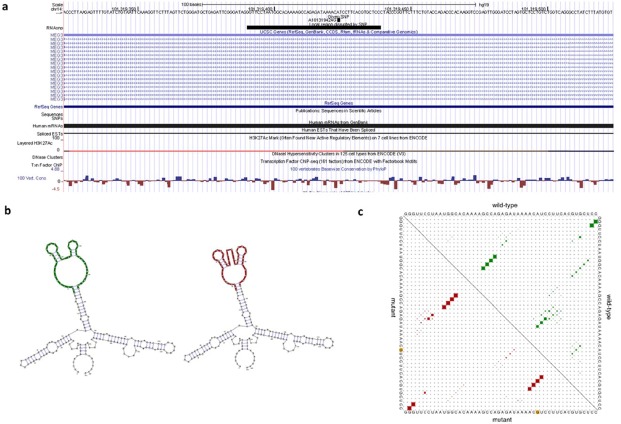
The predicting rs7158663 on *MEG3* secondary structure **a.** UCSC genome browser view for structure-disruptive rs7158663. **b.** Minimum free energy structures of rs7158663. **c.** Dot plot of the global structure.

We peformed an *in silico* analysis of rs7158663 on *MEG3* by RNAsnp. As shown in Figure 1, the RNAsnp predicted that rs7158663 G-A allele substitution leaded to a minimum free energy (MFE) change from −53.30 kcal/mol to −55.20 kcal/mol. Moreover, the base pair probabilities were different between rs7158663 G allele and A allele.

**Table 5:** Association analyses between *MEG3* rs7158663 genotypes and clinicalpathologic characteristics of cases

Variable	GG	GA/AA	OR (95%CI)^a^	*P*^a^
	*N* (%)	*N* (%)		
Site				
Colon	126 (24.4)	122 (23.6)	1.00	
Rectum	138 (26.7)	130 (25.2)	1.04 (0.73-1.46)	0.842
Stage				
I/II	126 (24.4)	125 (24.2)	1.00	
III/IV	138 (26.7)	127 (24.6)	1.10 (0.78-1.55)	0.601

a Adjusted for age, sex, and smoking status.

## DISCUSSION

We investigated the association of tagSNPs of *MEG3* with colorectal cancer risk in a population of Chinese. We demonstrated, for the first time, that rs7158663 in *MEG3* had a strong association with increased risk of colorectal cancer. In addition, the elevated risk was greater for subjects with age ≤ 60 and family history of cancer. These results suggested that rs7158663 may be a susceptibility SNP for colorectal cancer in Chinese populations.

Given *MEG3* is a lncRNA that plays critical roles in tumor cellular proliferation, migration and invasion [13, 15, 16], SNPs in *MEG3* may affect cell phenotypes and cause the risk of developing cancer. *MEG*3 is located on chromosome 14q32, a region proposed to contain putative tumor suppressors [17]. Although the regulation of *MEG3* and its precise mechanism of action in cancer are still well known, emerging evidence strongly demonstrates that *MEG3* functions as a novel lncRNA tumor suppressor [18]. In additional to colorectal normal tissue, *MEG3* is highly expressed in brain, lung, and liver [19]. Interestingly, we found that *MEG3* rs7158663 is correlated to significantly increased risk of colorectal cancer, which may be explained by the finds from Yin et al. [14]. They found that *MEG3* predicts a poor prognosis of colorectal cancer by influencing cell proliferation.

Until now, some studies have indicated that SNPs in lncRNAs could potentially impact various biological processes by influencing biological pathways. For example, Yang et al. first revealed the relationship between lncRNA *H19* and gastric cancer; they found an important role for *H19* variants in gastric cancer carcinogenesis [8]. Zhang et al. identified an allelic regulation of rs920778 on lncRNA *HOTAIR* expression, as well as associated with the development and progression of esophageal squamous cell carcinoma [20]. The novel function of rs920778 on *HOTAIR* was further confirmed in other cancer, including colorectal cancer [21], gastric cancer [22], and breast cancer [23]. The RNAsnp prediction revealed that rs7158663 in *MEG3* changed the folding structures of *MEG3*. Therefore, we speculate that rs7158663 could be a regulatory SNP, which regulate expression of *MEG3* and contribute to genetic susceptibility of colorectal cancer.

Several limitations of the present study should be mentioned. Firstly, these findings were based on genetic analysis of a single gene with a relatively small size of colorectal cancer patients, which need to be validated using an independent prospective clinical study. Secondly, our study was based on a hospital-based study; therefore, potentially important sources of selection bias may exist. Thirdly, diet is a major determinant of risk for colorectal cancer [24]; however, our study had incomplete information on dietary consumption. Further studies are needed to determine the association between dietary information and genetic variants in *MEG3*.

In summary, we identified a genetic susceptibility SNP rs7158663 for colorectal cancer in Chinese. The SNP rs7158663 in *MEG3* played a vital role in colorectal carcinogenesis. These data suggest that genetic variants in *MEG3* may serve as potential risk factor and targets for colorectal cancer therapy in the further.

## MATERIALS AND METHODS

### Study subjects

This study consisted of 518 colorectal cancer cases and 527 control subjects from the hospitals of Southeast University Medical College. All cases were patients newly diagnosed with histologically confirmed colorectal adenocarcinomas who were admitted to the hospitals. No patient had received radiotherapy or chemotherapy. Controls were frequency-matched with cases on age and sex who were recruited at the same time period. All controls were unrelated ethnic Han Chinese and these subjects had no history of cancer. All participants provided informed consent after the interview. This research protocol was approved by the Institutional Review Board of Southeast University Medical College.

### SNPs selection

We selected the tagSNPs of *MEG3* with the MAF > 0.05 in Han Chinese from the 1000 Genome Projects. As a result, six tagSNPs were selected using a pairwise Tagger method with *r*^2^ > 0.8 to capture other SNPs, rs10144253.

### Genotyping

Genomic DNA was extracted from peripheral blood using the TIANamp Blood DNA kit (Tiangen, China). Genotyping was performed by TaqMan SNP genotyping assay. Real-time TaqMan PCR and genotyping were conducted on an ABI 7500 real-time PCR System (Applied Biosystems, USA). The results of allelic discrimination were analyzed using SDS 2.4 software. For quality control, we included two negative controls (water) and two duplicates in each 96-well plate. Furthermore, about 3% of selected samples were repeated genotyping to confirm the results in a blind fashion.

### Statistical analysis

Tests for the Hardy-Weinberg equilibrium in cases and controls were performed by good-of-fit χ^2^ test. Student's t-test or χ^2^ test was used to assess the significance of any differences in frequency distributions of demographic variables and genotypes among cases and controls. We estimated the association between genotypes and colorectal cancer risk by odds ratios (ORs) and 95% confidence intervals (CIs) using the logistic regression. The ORs and 95%CIs were further adjusted by for age, sex, and smoking habits. All analyses were two-sided and *P* < 0.05 was considered significant. All statistical calculations were conducted with SPSS 13.0 software (SPSS Inc., Chicago, IL, USA).
